# Acceptability, feasibility, and efficacy of Internet cognitive behavioral therapy (iCBT) for pediatric obsessive-compulsive disorder: a systematic review

**DOI:** 10.1186/s13643-019-1166-6

**Published:** 2019-11-20

**Authors:** Lucía Babiano-Espinosa, Lidewij H. Wolters, Bernhard Weidle, Vivian op de Beek, Sindre A. Pedersen, Scott Compton, Norbert Skokauskas

**Affiliations:** 1 Department of Mental Health, Regional Centre for Child and Youth Mental Health and Child Welfare (RKBU), Norwegian University of Science and Technology (NTNU), Trondheim, Norway; 2 De Bascule, Academic Center for Child and Adolescent Psychiatry, Center of Expertise for OCD, Anxiety and Tics, Meibergdreef 5, 1105 AZ Amsterdam, The Netherlands; 3 Health, Library Section for Medicine and Health Sciences, Norwegian University of Science and Technology, Olav Kyrres gate 9, 7030 Trondheim, Norway; 4 Department of Psychiatry and Behavioral Sciences, Duke University School of Medicine, 2200 W Main St #340, Durham, NC 27705 USA

**Keywords:** eHealth, Obsessive-compulsive disorder, Cognitive behavioral therapy, Adolescent, Child

## Abstract

**Background:**

Obsessive-compulsive disorder (OCD) is a chronic mental health disorder characterized by recurring obsessions and compulsions affecting 1–3% of children and adolescents. Current treatment options are limited by accessibility, availability, and quality of care. New technologies provide opportunities to address at least some of these challenges. This paper aims to investigate the acceptability, feasibility, and efficacy of traditional cognitive behavioral therapy with Internet cognitive behavioral therapy (iCBT) for pediatric OCD according to Preferred Reporting Items for Systematic Reviews and Meta-Analyses (PRISMA) guidelines.

**Method:**

We searched EMBASE, Medline, PsycINFO, CENTRAL, LILACS, CINAHL, and Scopus. Results include articles from 1987 to March 2018. Main inclusion criteria were patients aged 4–18, primary diagnosis of OCD, and iCBT.

**Results:**

Of the 2323 unique articles identified during the initial search, six studies with a total of 96 participants met our inclusion criteria: three randomized controlled trials, one single-case multiple-baseline design, one open-label trial, and one case series. Four studies reported a significant decrease in OCD severity on the Children’s Yale-Brown Obsessive-Compulsive Scale (CY-BOCS) following iCBT, one study reported significant decrease in CY-BOCS scores for iCBT relative to waitlist, and the case series reported (some) symptom reduction in all participants. Six studies reported high rates of feasibility, and five studies reported good acceptability of iCBT.

**Conclusion:**

At present, evidence regarding acceptability, feasibility, and efficacy of iCBT for pediatric OCD is limited. Results are promising but need to be confirmed and refined in further research.

**Systematic review registration:**

PROSPERO CRD4201808587

## Background

Obsessive-compulsive disorder (OCD) is a disabling mental health disorder affecting between 1 and 3% of children and adolescents [1]. OCD is characterized by disturbing recurring thoughts (obsessions) and repetitive behaviors (compulsions) [1] and is associated with significant impairment [2] and reduced quality of life [3]. Without treatment, OCD has a chronic course in about 40–60% of those affected [4, 5].

Over the last three decades, OCD has moved from an almost untreatable, life-long psychiatric disorder to a highly manageable one. Two recent meta-analyses have supported cognitive behavioral therapy (CBT) as the first-line treatment for children and adults with OCD [6, 7] and two other meta-analyses reported larger effect sizes for CBT than for selective serotonin reuptake inhibitors (SSRIs) in pediatric OCD [8, 9]. While relapse is common after cessation of medication, treatment gains from CBT appear more stable [10]. Still, CBT for pediatric OCD has not reached its full potential, with response rates ranging between 40 and 65% [11, 12]. In addition, stigma about mental health treatment in general and OCD in particular, limited access to high-quality CBT, and the high costs of CBT may reduce treatment uptake [13]. Sixty to 90% of adults with OCD from Western countries and China are not seeking treatment for OCD [14].

New technologies and increased access to the Internet provide unique opportunities to address some of these challenges by offering more interactive, child-appealing [15], cost-effective [16], and more easily accessible therapies [17]. Illustrating this growing trend, the National Institute of Mental Health (NIMH) in the USA created the National Advisory Mental Health Council Workgroup on Opportunities and Challenges of Developing Information Technologies on Behavioral and Social Science Research [18]. Electronic and mobile health technologies are also included in the World Health Organization (WHO) Mental Health Action Plan 2013–2020 [19]. Internet cognitive behavioral therapy (iCBT) includes therapist-guided and automated interventions that are delivered using the Internet and information-technology based on cognitive behavioral therapy [20]. A recent systematic review indicated that Internet-based treatment programs for anxiety disorders and depression were generally well received by children and their parents [15]. These iCBT programs were effective in reducing anxiety symptoms, and some proved to be as effective as face-to-face interventions [15, 16, 21]. However, the effects on depression symptoms in adolescents and young adults (12–25 years) were small [16]. Previous meta-analysis has been published on iCBT for adult OCD showing good efficacy [22, 23]. To our knowledge, no systematic review has investigated the acceptability, feasibility, and efficacy of Internet cognitive behavioral therapy (iCBT) for pediatric OCD. The present systematic review aims to bridge this gap.

## Method

### Search strategy

The first paper about OCD treatment involving computer technology was published in 1987 [24]. This systematic review included studies published from 1987 to March 2018. The Cochrane database was assessed to ensure that no similar systematic review had been published. We searched the seven relevant databases: EMBASE, Medline, PsycINFO, CENTRAL, LILACS, CINAHL, and Scopus. The literature search involved a combination of thesaurus and free-text terms optimized to identify references containing three main concepts: “OCD,” “Internet technology-based therapy,” and “children or adolescents” Internet technology. (The exact keywords can be found in Additional file 2: study protocol.) V.B., S.P., and L.B.E. conducted the initial database research. L.W. and L.B.E independently filled the data collections forms that had been developed a priori by V.B. The data collection forms included (a) general information about the study (publication type, country of origin, funding), (b) study eligibility (inclusion criteria, sample details, study design, types of intervention, reasons to exclude), (c) study characteristics (aim, design, participants, outcomes), and (d) risk of bias assessment. L.W. and L.B.E. assessed article eligibility. In case of disagreement, consensus was reached through discussion with the other group members (B.W., N.S.). Relevant conference abstracts were searched manually to reduce potential limitations of the systematic database search. Finally, relevant Cochrane reviews, the WHO trials portal (ICTRP), ClinicalTrials.gov, and Google Scholar were searched to identify additional studies (see Additional file 2 for the study protocol).

### Inclusion and exclusion criteria

Inclusion and exclusion criteria were based on the “PICOS” [25] approach to review empirical studies: population, intervention, comparators, outcomes, and study design.

#### Inclusion criteria

Population:

- Children and adolescents aged 4–18
- Primary diagnosis of obsessive-compulsive disorder diagnosed by a psychologist or psychiatrist according to DSM or ICD criteria
- All treatment settings
- Any cultural background, ethnicity, and sex

Intervention:

- CBT with Internet technology components
- No restrictions on therapist involvement or additional treatment

Comparator:

- Studies with and without comparators
- No restrictions were set on comparators.

Outcome:

- Treatment acceptability refers to the degree to which an individual perceives a treatment protocol as appropriate, fair, and reasonable for a given population or problem and any acceptability test is accepted as an outcome [26].
- Feasibility refers to whether treatment works in practice and drop-outs are accepted as main outcome [26].
- Treatment efficacy refers to the capacity to improving health-outcomes. Children’s Yale-Brown Obsessive-Compulsive Scale (CY-BOCS) is accepted as the golden standard for its assessment [9].

For an overview of assessment instruments in this article, please see Additional file 3.

Study Design:

- Randomized controlled trial, blind trial, non-blind trial, adaptive clinical trial, non-randomized trial, interrupted time series design, cohort study, case-control study, and cross-sectional study published in English [27].

#### Exclusion criteria

Population:

- Adults
- Diagnosis of obsessive-compulsive disorder not determined by a qualified specialist (psychologist or psychiatrist) or not according to DSM or ICD criteria

Intervention:

- Other than CBT

Comparator:

- Studies with and without comparators are accepted.
- No restrictions were set on comparators.

Outcome:

- Not reporting on acceptability, feasibility, and efficacy.

Study Design:

- Qualitative study, commentary, correction, editorial letter (unless research letter reporting data), and single-case reports

## Results

### Search results

**Fig. 1:**
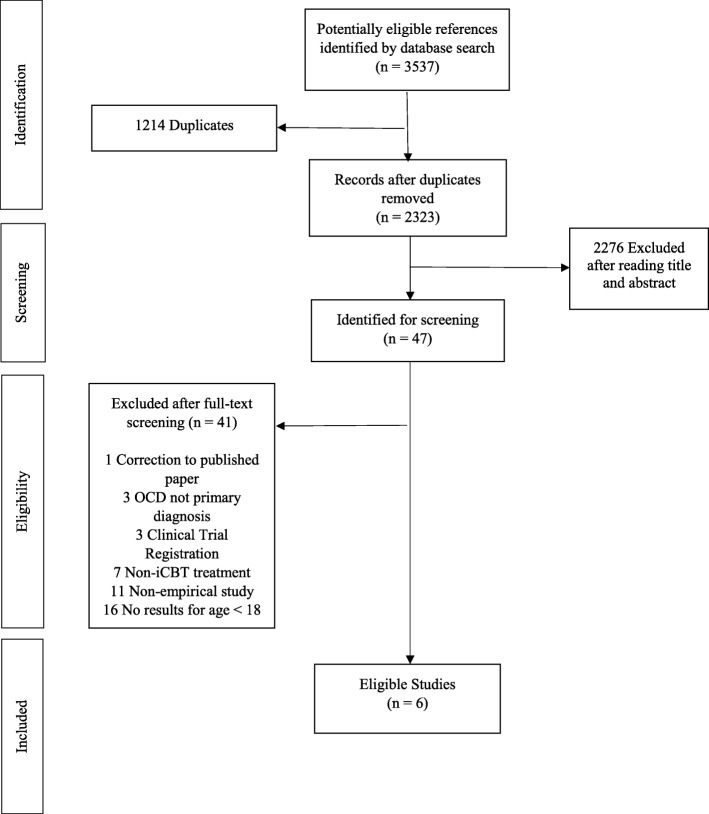
Flowchart

The initial search identified 3537 references. After removing 1214 duplicates, the search resulted in 2323 references. Of these, 2276 references were excluded after screening titles and abstracts, resulting in 47 references that were thoroughly screened. Forty-one from these 47 references were removed due to conflicts with selection criteria. Finally, six original studies were included in this systematic review (see Fig. 1 for flow diagram).

The reviewed studies included a total of 96 participants (47 girls, 49 boys) with a mean age ranging from 6.5 [28] to 14.4 years [29] (Table 1). Two studies included children aged 4–8 years [28, 30], other studies included children and adolescents aged 7–17 years [29, 31, 32, 34]. The studies were conducted in North America [28, 30, 34], Australia [31], and Europe [29, 32]. There were three randomized controlled trials (RCTs) [29, 30, 34], one open trial [32], one single-case non-concurrent multiple-baseline design [31], and one case series [28] (Table 1). Two studies recruited families seeking treatment in outpatient clinics [28, 30]. Three studies [29, 32, 34] recruited participants from outpatient clinics and used advertisements in local newspapers, websites, and radio, and one study [31] combined advertisements in local newspapers with referrals from general practitioners.

**Table 1:** Overview of the eligible studies on iCBT for pediatric OCD

Reference	Study design	Control group	Participants	Parent involvement	Communication methods	Therapist involvement	Intervention		Outcome	
							Intervention contents	Intervention duration	Primary outcomes	Time of assessment
Comer et al. (USA) [28]	Case series	Not Applicable	Children aged 4-8 (*M* = 6.5; SD = 0.9) 3 boys 2 girls	(a) Parents were trained as coaches, (b) treatment addresses parental accommodation of child symptoms, and (c) treatment had an exposure component for parents.	Video-teleconference sessions	Regular contact through video-teleconference	“Internet-delivered Family-based -CBT”: -Video teleconferencing -Interactive computer games, feeling thermometer, exposure hierarchy and exercises	12 sessions in 14 weeks	Treatment efficacy, feasibility, and acceptability	Baseline, post-treatment
Comer et al. (USA) [30]	RCT	Family-based CBT delivered in clinic	Children aged 4–8 (*M* = 6.7; SD = 1.3) 6 boys 5 girls	(a) Parents were trained as coaches, (b) treatment addresses parental accommodation of child symptoms, and (c) treatment had an exposure component for parents.	Video-teleconference sessions	Regular contact through video-teleconference	“Internet-delivered Family-based -CBT”: -Video teleconferencing -Interactive computer games, feeling thermometer, exposure exercises and hierarchy	12 sessions in 14 weeks	Treatment efficacy, feasibility, and acceptability	Baseline, post-treatment, 6-month follow up
Farrell et al. (Australia) [31]	Single-case, non-concurrent multiple-baseline design	Not Applicable	Adolescents aged 11–16 (*M* = 13.6; SD = 1.8) 6 boys 4 girls	Parents were involved in education session, at the end of their child’s intensive face-to-face sessions, and during all e-therapy maintenance sessions.	One face-to-face education session, two intensive face-to-face CBT sessions, therapy maintenance sessions via video-teleconferencing	Regular contact through video-teleconferencing	Two intensive face-to-face CBT sessions followed by e-therapy maintenance	Psychoeducation and 2 intensive CBT sessions during 3 weeks, followed by a 3-week therapy maintenance program	Treatment efficacy and feasibility	Pre-intervention, weekly assessments during 1- or 2-week baseline period, post-CBT, 1-month follow up (after e-therapy), 6-month follow up
Lenhard et al. (Sweden) [32]	Open trial	Not Applicable	Adolescents aged 12–17 (M = 14.4; SD = 2.6) 8 boys 13 girls	Parents participated in treatment through parent-specific chapters, with varying degrees of parental involvement depending on the child’s age.	“Internet Project for Children”: a self-help protocol through an Internet platform containing texts, films, animations and exercises; telephone calls or messages	Occasional contact through telephone calls or messages to a therapist	“Internet Project for Children” Internet platform for educative texts, films, and exercises	12 treatment chapters in 12 weeks	Treatment efficacy, feasibility, and acceptability	Baseline, 3-month, post-treatment, 6-month follow up
Lenhard et al. (Sweden) [29, 33]	RCT	Waitlist	Adolescents aged 12-17 (*M* = 14.2; SD = 1.7) 16 boys 17 girls	Parents participated in the treatment through parent-specific chapters, with varying degrees of parental involvement depending on the child’s age	“Internet Project for Children”: a self-help protocol through an Internet platform containing texts, films, animations and exercises; telephone calls or messages. Smartphone application support for ERP exercises	Occasional contact through telephone calls or messages to a therapist	“Internet Project for Children” Internet platform for educative texts, films, and exercises	12 treatment chapters in 12 weeks	Treatment efficacy, feasibility, and acceptability	Baseline, post-treatment, 3-month follow up
Storch et al. (USA) [34]	RCT	Waitlist	Children and adolescents between 7 and 16 (*M* = 11.1; SD = 2.6) 10 boys 6 girls	Parents were instructed on coaching their child through exposure.	Video-teleconference sessions, email	Regular contact through video-teleconference	Web camera-delivered cognitive behavioral therapy (video-teleconference)	14 sessions in 12 weeks	Treatment efficacy, feasibility, and acceptability	Baseline, post-treatment

*RCT* randomized controlled trial, *CBT* cognitive behavioral therapy, *iCBT* Internet cognitive behavioral therapy

Table 1 provides a description of the iCBT interventions. All studies included psychoeducation about OCD for the patients and their parents, as well as information about the treatment procedure. Exposure and response prevention (ERP) and cognitive interventions were key components in all procedures. All studies employed experienced clinicians [28–32, 34], and five studies also used psychology students as therapists [28–30, 32, 34]. Weekly supervisions for the therapists were performed to ensure the standards of treatment procedure [28–32, 34].

Two studies by Comer and colleagues and one by Farrell and colleagues provided a specific training for their therapists [28, 30, 31]. Therapist involvement varied from minimal (with occasional indirect contact via messages or phone) [29, 32] to substantial (via frequent video-teleconferencing) [34]. Parents were actively involved in the treatment process in all studies.

Two studies by Comer et al., and one by Farrell et al., provided a specific training for their therapists [28, 30, 31]. Therapist involvement varied from minimal (with occasional indirect contact via messages or phone) [29, 32] to substantial (via frequent video-teleconferencing) [34]. Parents were actively involved in the treatment process in all studies. Comer et al. first performed a case series [28] and subsequently an RCT [30] using the same iCBT concept. In the RCT, they compared 14 weeks face-to-face family-based CBT with family-based iCBT treatment. The iCBT included video-teleconferencing and interactive computer games that were added to enhance the children’s understanding of the treatment concepts. Lenhard et al. [29, 32] evaluated “Internet Project for Children”, an iCBT intervention delivered via an Internet platform with psychoeducational texts, films, animations, and exercises (12 sessions), in an open trial [32] followed by an RCT [29]. During this treatment, patients had irregular asynchronous contact with a therapist through messages and occasional telephone calls. Storch et al. [34] compared 14 CBT sessions delivered via video-teleconferencing with a waitlist control. Their iCBT program followed the Pediatric OCD Treatment Study (POTS) protocol with some adaptations, such as using email to send homework instructions [35]. Farrell et al. [31] evaluated a 6-week intensive treatment program combining iCBT and face-to-face CBT. This intervention included a 1-h face-to-face psychoeducation session and two face-to-face intensive exposure and response prevention (ERP) sessions in 2 weeks, followed by a 3-week maintenance program delivered via video-teleconferencing.

### Acceptability

Acceptability was examined using validated self-report questionnaires, such as the Client Satisfaction Questionnaire (CSQ) and the Working Alliance Inventory (WAI) [28, 30], and several newly developed questionnaires (Tables 2 and 3) [29, 30, 32].

**Table 2:** Outcomes of acceptability, feasibility, and efficacy (non-randomized controlled trials)

Reference	Measure	Pre-treatment M (SD)	Post-treatment M (SD)	Within group Significance Pre-post	Within group Size effect (d) Pre-post	Follow-up M (SD)
Comer et al. (USA) [28]	**Efficacy**					
	CY-BOCS	24.2 (5.2) ^c^	17.4 (5.9) ^c^	Not Reported	2.54	Not Applicable
	ADIS-IV-C/P (OCD CSR)	6.2 (1.1) ^c^	4.0 (1.4) ^c^	Not Reported	5.88	Not Applicable
	CGAS	51.8 ^d^	58.6 ^d^	Not Reported	2.87	Not Applicable
	CGI-S	5.2 ^d^	3.6 ^d^	Not Reported	Not Reported	Not Applicable
	CGI-I	Not Applicable	2.2 (0.8) ^c^	Not Reported	Not Reported	Not Applicable
	**Acceptability**					
	CSQ-8 (First Item)	Not Applicable	All mothers rated quality as “Excellent”	Not Applicable	Not Applicable	Not Applicable
	**Feasibility**					
	Treatment dropout	Not Applicable	Dropout = 0	Not Applicable	Not Applicable	Not Applicable
Farrell et al. (Australia) [31]	**Efficacy**					
	CYBOCS	29.1 (4.2)	14.8 (7.7)	p< 0.001	2.09	11.8 (8.9) ^b^
	CY-BOCS-SR (Parent)	24.1(3.3)	12.9 (7.3)	p< 0.001	1.94	11.5 (9.5) ^b^
	ADIS-IV-C/P (OCD CSR)	6.6 (0.5)	3.5 (2.0)	p< 0.001	2.28	3.3 (1.9) ^b^
	NIMH GOCS	10.7 (1.8)	6.3 (3.1)	p< 0.005	1.36	5.8 (3.6) ^b^
	CGI-S	5.6 (0.5)	3.1 (1.5)	p< 0.001	2.25	2.7 (1.6) ^b^
	CDI-S	13.6 (10.9)	10.3 (7.9)	p< 0.05	0.34	Not Reported
	MASC	83.6 (35.0)	60.1 (26.1)	p= n.s.	0.76	Not Reported
	PEDSQL	35.3 (12.1)	18.5 (14.9)	p< 0.05	1.23	Not Reported
	**Feasibility**					
	Treatment dropout	Not Applicable	Dropout = 0	Not Applicable	Not Applicable	Not Applicable
Lenhard et al. (Sweden) [32]	**Efficacy**					
	CY-BOCS	21.3 (3.5)	12.1 (4.5)	p< 0.001	2.29	8.8 (5.1) ^a^ 9.1 (6.4) ^b^
	ChOCI –symptom Parent	12.4 (6.8)	6.5 (5.1)	p< 0.001	0.94	5.3 (5.6) ^a^ 4.5 (4.3) ^b^
	ChOCI –impairment Parent	24.9 (7.0)	17.8 (10.0)	p< 0.001	0.79	12.4 (8.1) ^a^ 11.5 (6.4) ^b^
	ChOCI –symptom child	13.6 (8.7)	6.4 (6.6)	p< 0.001	0.92	5.3 (6.7) ^a^ 5.0 (6.6) ^b^
	ChOCI – impairment child	22.6 (8.1)	11.6 (6.3)	p< 0.001	1.51	9.9 (8.9) ^a^ 10.4 (9.1) ^b^
	COIS-R Parent	25.3 (16.1)	16.8 (17.2)	p< 0.05	0.45	13.0 (15.7) ^a^ 13.9 (15.0) ^b^
	COIS-R Child	17.3 (15.5)	6.6 (7.9)	p< 0.001	0.88	5.2 (8.4) ^a^ 6.0 (9.0) ^b^
	CGI-I	Not Applicable	52% “Much Improved” or “Very Much Improved”	Not Applicable	Not Applicable	71 % “Much Improved” or “Very Much Improved” ^a b^
	CGAS	56.1 (6.3)	71.5 (9.3)	p< 0.001	-1.94	74.0 (9.0) ^a^ 73.5 (9.7) ^b^
	CDI-S	9.6 (1.4)	9.9 (1.2)	p= n.s.	-0.19	2.5 (2.7) ^a^ 2.2 (2.1) ^b^
	FAS	14.6 (8.4)	9.6 (7.1)	p<0.05	0.60	6.9 (8.1) ^a^ 6.5 (6.9) ^b^
	SDQ Parent	12.0 (6.7)	10.3 (6.3)	p= n.s.	0.29	10.3 (6.6) ^a^ 9.7 (6.4) ^b^
	SDQ child	13.5 (5.5)	10.6 (4.0)	p= n.s.	0.61	10.7 (4.2) ^a^ 10.5 (4.8) ^b^
	SCAS without OCD Parent	25.2 (15.7)	16.0 (13.5)	p<0.001	0.63	16.4 (12.2) ^a^ 15.7 (14.1) ^b^
	SCAS OCD Child	9.1 (5.0)	4.1 (3.4)	p<0.001	1.17	2.9 (3.8) ^a^ 3.3 (4.0) ^b^
	SCAS without OCD Child	30.4 (16.9)	20.2 (13.5)	p<0.001	0.67	18.9 (14.0) ^a^ 18.3 (14.2) ^b^
	**Acceptability**					
	Not Applicable	Not Applicable	Not Applicable	Not Applicable	Not Applicable	Not Applicable
	**Feasibility**					
	Treatment dropout	Not Applicable	8.3/12 Chapters completed by patients (3.0)	Not Applicable	Not Applicable	Not Applicable
			4.7/5 Chapters completed by parents (0.8)			

^a^for 3 months; ^b^ for 6 months; ^c^ mean and standard deviation calculated by the reviewer; ^d^ standard deviation and data required for calculating SD not provided

**Table 3:** Outcomes of acceptability, feasibility, and efficacy (randomized controlled trials)

Reference	Measure	Pre-treatment M (SD)	Post-treatment M (SD)	Between groups Significance (WL-CBT)	Between groups Significance (iCBT-CBT)	Within group Significance PRE-POST	Between groups Effect size (d)	Follow-up M (SD)
Comer et al. (USA) [30]	**Efficacy**							
	CY-BOCS	22.9 (4.1)	14.9 (7.3)	Not Applicable	p=n.s	Not Reported ^c^	0.09	11.8 (9.5) ^b^
	ADIS-IV-C/P (OCD CSR)	5.1 (0.8)	3.4 (1.2)	Not Applicable	p=n.s	Not Reported ^c^	0.24	2.4 (2.6) ^b^
	CGI-S	4.9 (0.7)	3.2 (1.5)	Not Applicable	p=n.s	Not Reported ^c^	-0.06	2.6 (2.5) ^b^
	CGAS	48.0 (8.0)	61.4 (12.0)	Not Applicable	p=n.s	Not Reported ^c^	-0.06	66.6 (15.9) ^b^
	FAS	29.5 (7.8)	19.5 (9.7)	Not Applicable	p=n.s	Not Reported ^c^	0.56	15.6 (14.2) ^b^
	**Acceptability**							
	CSQ-8	Not Applicable	Mother 28.6 (4.5)	Not Applicable	p=n.s.	Not Applicable	Not Applicable	Not Applicable
	WAI	Not Applicable	Mother 223.5 (34.8) Therapist 226.1 (32.9)	Not Applicable	p=n.s. p=n.s.	Not Applicable	Not Applicable	Not Applicable
	**Feasibility**							
	Treatment drop-out	Not Applicable	Dropout = 1	Not Applicable	Not Applicable	Not Applicable	Not Applicable	Not Applicable
Lenhard et al. (Sweden) [29, 33]	**Efficacy**							
	CY-BOCS	23.0 (4.3)	17.0 (6.3)	p<0.001	Not Applicable	Not Reported	0.69	14.2 (5.9) ^a^
	ChOCI-R child	24.5 (6.7)	20.0 (7.8)	p=0.014	Not Applicable	Not Reported	0.64	19.3 (8.3) ^a^
	ChOCI-R parent	24.4 (7.6)	19.3 (9.9)	p=0.012	Not Applicable	Not Reported	0.59	17.7 (8.7) ^a^
	CDI	4.7 (3.4)	4.6 (4.0)	p=n.s.	Not Applicable	Not Reported	-0.01	4.7 (4.2) ^a^
	SCAS parent	10.7 (5.8)	8.3 (5.9)	p=0.004	Not Applicable	Not Reported	0.67	8.4 (5.6) ^a^
	SCAS child	12.9 (6.4 )	11.4 (7.4)	p=n.s.	Not Applicable	Not Reported	0.27	10.4 ( 6.4) ^a^
	EWSAS child	14.8 (9.2)	12.8 (9.7)	p=n.s.	Not Applicable	Not Reported	0.27	10.7 (9.1) ^a^
	EWSAS parent	16.1 (8.6)	11.4 (8.5)	p<0.001	Not Applicable	Not Reported	0.43	11.1 (9.2) ^a^
	FAS	15.8 (11.3)	11.2 (9.2)	p=0.003	Not Applicable	Not Reported	0.54	10.6 (10.2) ^a^
	**Acceptability**							
	Self-made questionnaire	Not Applicable	46% of the patients were satisfied with internet- delivered format, 50% would have liked to meet a clinician 4% would have preferred face-to-face treatment Patients’ treatment rating: 32% Very Good, 32% Good 36% Ok, 0% Bad	Not Applicable	Not Applicable	Not Applicable	Not Applicable	Not Applicable
	**Feasibility**							
	Treatment drop-out	Not Applicable	8.5/12 chapters completed (2.9) Dropout = 1	Not Applicable	Not Applicable	Not Applicable		Not Applicable
Storch et al. (USA) [34]	**Efficacy**							
	CY-BOCS	25.4 (3.6)	11.1 (10.5)	p<0.001	Not Applicable	Not Reported	1.36	11.3 (9.4) ^a^
	COIS Parent	42.8 (23.4)	16.8 (24.5)	p=0.005	Not Applicable	Not Reported	0.99	Not Reported
	COIS child	38.8 (24.1)	16.1 (19.0)	p=0.03	Not Applicable	Not Reported	0.46	Not Reported
	CGI-S	3.8 (0.9)	1.6 (1.8)	p<0.001	Not Applicable	Not Reported	1.48	1.4 (1.3) ^a^
	CGI-I	Not Applicable	13/16 participants (81%) responder (a ≥30% reduction in CY-BOCS score and a CGI-I score of 1 or 2)	p<0.001	Not Applicable	Not Reported	Not Applicable	Not Reported
	CDI	8.9 (6.7)	7.5 (8.0)	p=n.s.	Not Applicable	Not Reported	0.43	Not Reported
	MASC	39.9 (14.8)	33.4 (14.8)	p=n.s.	Not Applicable	Not Reported	0.46	Not Reported
	FAS	25.7 (8.6)	16.1 (13.9)	p=0.003	Not Applicable	Not Reported	0.37	Not Reported
	**Acceptability**							
	PWA	Not Applicable	19.4 (1.3) Parents’ Satisfaction	Not Applicable	Not Applicable	Not applicable	Not applicable	Not Applicable
	**Feasibility**							
	Treatment drop-out	Not Applicable	Dropout = 2	Not Applicable	Not Applicable	Not applicable	Not applicable	Not Applicable

In the open trial by Lenhard et al., [32] treatment acceptability was evaluated in adolescents and at least one parent. The Internet Project for Children was rated as good or very good by the families [32]. In the following RCT, only adolescents’ views were assessed [29]. Results showed that 46% of the adolescents were satisfied with the Internet Project for Children, 50% were satisfied most of the time but would have liked to meet a clinician occasionally (contact with a therapist was established through e-mail messages and phone calls only), and 4% would have preferred face-to-face treatment [29]. Other studies assessed parents’ views only [28, 34]. In both studies by Comer et al., all mothers [28, 30] reported good alliance with the therapist and that they were satisfied with the treatment. Storch et al. [34] reported very high satisfaction with treatment rated by parents. One study did not report on acceptability [31].

### Feasibility

We examined treatment feasibility by documenting drop-out from treatment, which ranged from none [28, 31] to two patients across all studies [34] (Tables 2 and 3). Altogether, 4.2% (four patients) dropped-out from all treatments. No participants dropped out from treatment in the case series study by Comer et al. [28], nor in the study by Farrell et al. [31]. One patient dropped out in the RCT by Comer et al. [30] (after session one; reason not reported). In Storch and colleagues’ study [34], two participants withdrew from treatment due to a lack of perceived benefit. In the RCT from Lenhard et al. [29], one treatment drop-out was reported.

### Efficacy

All studies used the Children’s Yale-Brown Obsessive-Compulsive Scale (CY-BOCS) [36] to assess the severity of OCD symptoms (Tables 2 and 3). Four studies reported a statistically significant decrease in CY-BOCS scores from pre- to post-treatment [30–32, 34]. Comer et al. [28] reported that three of the five participants had a post-treatment CY-BOCS score < 16 (clinical cut off), two other participants showed minimal improvement. Lenhard et al. reported a significant improvement in CY-BOCS score after iCBT [29]. Comer et al. [30] reported no significant difference between face-to-face CBT and iCBT.

Storch et al. [34] reported more than a half reduction (56.1%) in OCD symptoms on the CY-BOCS following iCBT. Lenhard et al. [29, 32] reported an average reduction of 41% and 26%^1^ in CY-BOCS scores following iCBT in their open trial and RCT, respectively. Results from the study by Farrell et al. [31] showed 49%^1^ symptom reduction at post-treatment. Comer et al. reported a 35%^1^ and 28%^1^ average reduction in CY-BOCS scores at post-treatment in their RCT [30] and case series [28], respectively. In addition, four studies reported improvements at post-treatment on the Children’s Global Assessment Scale (CGAS) [37] and the Clinical Global Impression Scale (CGI) [38] (Tables 2 and 3, see Additional file 3 for assessment glossary) [28, 30, 31, 34].

In their open trial, Lenhard et al. reported significant improvement at 3-month follow-up, which was maintained at 6-month follow-up [32]. In their RCT, participants continued to show significant improvement from post-treatment to 3-month follow-up [29]. Comer et al. reported significant improvement from pre-treatment to 6-month follow-up [30]. Farrell et al. reported 8 of 10 children in “reliable change” with at least 8.33 points in symptoms improvement on the CY-BOCS at post-treatment and 6-month follow-up (Tables 2 and 3) [31].

### Risk of bias

As recommended by the Cochrane Collaboration, we used the Cochrane Collaboration’s tool to assess risk of bias (low, unclear, or high-risk) among the eligible studies [39]. Overall results showed some risk of bias [39]. This was mainly due to the fact that even in the studies where a random generator was used to allocate participants to treatment condition, the need for the use of devices in the experimental condition (iCBT treatment), may be problematic for blinding participants to treatment condition [29, 30, 34]. As a consequence of this, there may be an unclear risk bias in the blinding of outcomes category [28–32, 34] (Table 4).

**Table 4:** Risk of bias assessment

		Random sequence generation bias (selection bias)	Allocation concealment (selection bias)	Blinding of participants and personnel (performance bias)	Blinding of outcome assessment (detection bias) Patient-reported outcomes	Blinding of outcome assessment (detection bias) Evaluator-reported outcomes	Incomplete outcome data (attrition bias) Post-treatment	Incomplete outcome data (attrition bias) Follow-up	Selective reporting (reporting bias)
Comer et al. (USA) [28]	Non-RCT	Not applicable	Not applicable	Not applicable	Unclear risk	Low risk	Low risk	Not applicable	Low risk
Comer et al. (USA) [30]	RCT	Low risk	Low risk	Unclear risk	Unclear risk	Low risk	Low risk	Low risk	Unclear risk
Farrell et al., 2016 (Australia) [31]	Non-RCT	Not applicable	Not applicable	Not applicable	Unclear risk	Low risk	Low risk	Low risk	Low risk
Lenhard et al., 2014 (Sweden) [32]	Non-RCT	Not applicable	Not applicable	Not applicable	Unclear risk	Unclear risk	Low risk	Low risk	Low risk
Lenhard et al., 2017 (Sweden) [29, 33]	RCT	Low risk	Low risk	Unclear risk	Unclear risk	Low risk	Low risk	Low risk	Low risk
Storch et al., 2011 (USA) [34]	RCT	Low risk	Low risk	Unclear risk	Unclear risk	Unclear risk	Low risk	Low risk	Low risk

## Discussion

To our knowledge, this is the first systematic review on the acceptability, feasibility, and efficacy of iCBT for pediatric OCD. We identified six eligible studies involving four different iCBT interventions for pediatric OCD with a total number of 96 subjects.

The last decade has seen a substantial increase in e-mental health development and research [40]. The low number of eligible studies for pediatric OCD is in striking contrast to the rising use of Internet and mobile devices all over the world [41], their extensive use during childhood and adolescence [42], and the rising interest in iCBT [19]. There are more studies on iCBT in other populations than pediatric OCD. In 2015, a meta-analysis on iCBT for adult OCD included eight RCTs (*N* = 420) and reported no significant difference in efficacy between iCBT and face-to-face cognitive behavioral therapy [22]. In 2016, another meta-analysis included 18 studies and results showed large effect sizes for remote treatment for OCD in adults [23]. These results are in line with the results that we found in the present review. Furthermore, a recent meta-analysis on smartphone applications for depressive symptoms in adults [43] identified 18 RCTs assessing 22 smartphone applications, compared to only one smartphone application in our review [29]. Results of the present review showed that high treatment acceptability was reported in the five studies where acceptability was assessed [28–30, 32, 34]. However, different assessment tools were used (i.e., CSQ-8, PWA, WAI, self-developed questionnaires), and acceptability assessment tools were not always standardized or validated [29, 32]. Albeit opinions regarding treatment can differ considerably between respondents, some studies assessed only mothers’ acceptance of the treatment [28, 30], some studies reported parents’ acceptance (not specifying which parent) [32, 34], some studies assessed working alliance evaluated by the therapist [30], and only two studies assessed children’s acceptance [29, 32]. Although acceptability was generally rated to be high in the study by Lenhard et al., [29] where therapist contact consisted of occassional e-mail, messages, and phone calls, half of participants reported that they would have liked to meet with a clinician occasionally, indicating that face-to-face therapist contact was an unmet need for part of this sample. In general, the findings in the present review regarding treatment acceptance are in line with the high acceptance of iCBT found for children with depression and anxiety [15]. Overall, thus far systematic reviews about Internet interventions for pediatric anxiety, depression, and internalizing problems have focused mainly on efficacy, and acceptability is generally under-reported [16, 21, 44].

Based on the low number of treatment drop-outs, ranging from none [28, 31, 32] to two individuals [29, 30, 34], feasibility was found to be high in all eligible studies. This is in line with two systematic reviews that reported good feasibility of Internet-assisted delivery of CBT for childhood anxiety and of web-based interventions for youth with internalizing problems [15, 44]. However, these results should be interpreted with caution due to the small samples of the included studies.

All studies reported favorable effects of iCBT on OCD symptoms. The reported efficacy of iCBT in the reviewed studies ranged between 26%^1^ [29] and 56%^1^ OCD symptom reduction [34]. A possible explanation for the variety in treatment effect is that development and application of iCBT programs are driven by different strategies. One strategy aims to overcome geographic barriers [34], other studies seek to improve limited response rates of conventionally delivered face-to-face CBT [28, 30, 31, 34], while another strategy aims to offer low-cost and easily accessible autonomous treatment programs [29, 32]. The heterogeneous results regarding efficacy should be interpreted according to the scope of the intervention.

Preliminary results indicate that treatment gains are maintained over time (3–6 months) [30–32]. While there is some evidence that treatment gains from face-to-face CBT on pediatric OCD are maintained at 1-year follow up [10], evidence concerning the sustainability of treatment results of iCBT is currently very limited.

None of the eligible studies reported a worsening of symptoms or any other treatment-related adverse events during iCBT. These results tentatively suggest that iCBT is a safe treatment. However, the spiraling growth of non-evidence-based e-health applications with poor guidance for users on how to make their choice causes concern [45]. Several potentially harmful effects, for example, regarding Internet security, confidentiality issues, and patient safety [46] were not assessed. This is a serious risk. A systematic review of Huckvale et al. [46] discovered systematic gaps for data security in 89% of the accredited health apps.

The main limitation of the current systematic review is the low number of eligible studies and their small samples [28–32, 34]. The six eligible studies came from three different continents (North America, Europe, and Australia) representing some cultural diversity although all belong to western cultures. As a result to our wide acceptance criteria, internal validity might be threatened [47]. There was a wide range of differences among the interventions, including the format of the intervention, the kind of Internet technology that was used, the length of treatment, and the amount of therapist contact. These differences make it difficult to draw an overall conclusion regarding the use of iCBT for pediatric OCD. However, the use of wide criteria made it possible to provide a complete overview of the state of the art in this field. In addition, wide inclusion criteria strengthened the external validity of this review, since the results show a realistic picture of the variety of iCBT treatments for pediatric OCD. A meta-analysis was not performed due to the small samples in the included studies, the low number of RCTs, and heterogeneity among treatments. Among the RCT’s, two were superiority trials with waitlist as control group [29, 34], and one was a non-inferiority trial with traditional face-to-face CBT as control group [30]. The two superiority trials [29, 34] examined very different iCBT treatments. One treatment consisted of a self-help program with minimal contact with the therapist [29]. The other treatment was based on regular contact with a therapist through video-teleconferencing [34]. These treatments aim to meet different needs for different patients. For these reasons, we believe that the results should be considered into the context of the treatments. Strengths of the present systematic review include the use of PRISMA guidelines to summarize and discuss the current state of acceptability, feasibility, and efficacy of iCBT for pediatric OCD (Additional file 1). These findings have importance for future directions. They do also raise questions requiring further research.

iCBT includes potential benefits offering CBT in a format that allows for reduced stigma and more widely available and accessible care. In addition, to meet the young patients in their area of expertise and using their “language” and way of cultural expression may enhance motivation for and adherence to the treatment program, which may contribute to more effective treatment and a reduced number of treatment drop-out. Studies exploring cost-effective and easy accessible autonomous treatment programs with minimal therapist contact are highly interesting in the scope of a stepped care model, allowing to differentiate between patients who benefit from this type of low-cost treatments and those who need therapist-delivered CBT [29, 32, 33]. In addition, intensive treatments, that could be delivered in varying formats, may be needed for other patients. A broader understanding about which format and amount of intervention works best for whom may lead to better outcomes and reduced societal costs. Future studies could focus on this question, assessing how different iCBT formats can augment traditional CBT by meeting the individual needs of patients. In addition, we need to know whether treatment gains obtained from these interventions will be maintained over time. It is also essential to assess and monitor potential adverse effects, for example, regarding Internet security, confidentiality issues, patient safety, and encryption. The use of smartphones, video-games, or health wearable trackers has the potential to both address barriers for treatment by adapting the therapy to the modern every-day life of the patient and to provide new possibilities for improved cost-effectiveness. However, the currently available scientific-evidence must improve substantially to enable the broader use of these new technologies.

## Conclusion

Although e-mental health development and research have increased substantially over the last 10 years, the currently available evidence-base for iCBT programs for pediatric OCD is limited. The results in this systematic review indicate that iCBT can be a feasible and acceptable treatment. Available limited evidence supports the use of i-tools to enhance ERP exercises and overcome barriers to treatment. However, replication studies with bigger samples are needed, along with studies testing which modalities and components for iCBT are most effective for whom.

## Supplementary information

**Additional file 1.** PRISMA 2009 checklistR1.

**Additional file 2.** Study protocol.

**Additional file 3.** Overview of assessment instruments.

## Data Availability

All papers available online: systematic review

## Abbreviations

CBT: Cognitive behavioral therapy
CENTRAL: The Cochrane Central Register of Controlled Trials
CGAS: Children’s Global Assessment Scale
CGI: Clinical Global Impression Scale
CINAHL: Cumulative Index of Nursing and Allied Health Literature
CSQ-8: The Client Satisfaction Questionnaire-8
CY-BOCS: Children’s Yale-Brown Obsessive-Compulsive Scale
DSM: The Diagnostic and Statistical Manual of Mental Disorders
EMBASE: Excerpta Medica dataBASE
ERP: Exposure and response prevention
iCBT: Internet cognitive behavioral therapy
ICD: International Statistical Classification of Diseases and Related Health Problems
ICTRP: International Clinical Trials Registry Platform
LILACS: Latin American and Caribbean Health Sciences Literature
NIMH: National Institute of Mental Health
OCD: Obsessive Compulsive Disorder
PICOS: Population, intervention, comparators, outcomes, and study design
PRISMA: Preferred Reporting Items for Systematic Reviews and Meta-Analyses
PWA: Parent Working Alliance
RCT: Randomized controlled trial
SSRIs: Selective serotonin reuptake inhibitors
WAI: Working Alliance Inventory
WHO: World Health Organization

## Footnotes

1 Percentages based on group differences between mean pre- and mean post-treatment score.
