# Supplementary material for: Acceptability, feasibility, and efficacy of Internet cognitive behavioral therapy (iCBT) for pediatric obsessive-compulsive disorder: a systematic review
Source: Syst Rev. 2019 Nov 20;8:284. doi: 10.1186/s13643-019-1166-6 (PMC6864940; doi:10.1186/s13643-019-1166-6)
Supplement: Supplementary file 2 — Additional file 2. Study protocol. [file 13643_2019_1166_MOESM2_ESM.docx]

# Additional File 2: Study protocol

Acceptability, Feasibility and Efficacy of Internet Cognitive Behavioral Therapy (iCBT) for Pediatric Obsessive-Compulsive Disorder: A Systematic Review

## Protocol information

Authors

L. Babiano-Espinosa ^a^, L.H. Woltersa,^c^, B. Weidle^a^, V. op de Beek ^a^, S.A. Pedersen ^b^, S. Compton ^d^, N. Skokauskas ^a^.

^a^Norwegian University of Science and Technology (NTNU,) Department of Mental Health, Regional Centre for Child and Youth Mental Health and Child Welfare (RKBU) Trondheim, Norway.

^b^Norwegian University of Science and Technology (NTNU,) Library Section for Medicine and Health Sciences, Trondheim, Norway.

^c^De Bascule, Academic Center for Child and Adolescent Psychiatry, Center of Expertise for OCD, Anxiety and Tics, Amsterdam, the Netherlands.

^d^Duke University School of Medicine, Department of Psychiatry and Behavioral Sciences, Durham, North Carolina.

Contact person

Lucía Babiano-Espinosa

Dates

| Preliminary searches | July 2016 |  |
| --- | --- | --- |
| Piloting of the study selection process | July 2016 |  |
| Formal screening of search results against eligibility criteria | March 2017 |  |
| Data extraction | March 2017 |  |
| Risk of bias assessment | April 2017 |  |
| Data analysis | April 2017 |  |
| Data update | March 2018 |  |
| Submitted | July 2019 |  |

History

Protocol first published: not previously published
Review first published: not previously published

## Introduction on the protocol

Description of the condition

Obsessive-compulsive disorder (OCD) is characterized by intrusive thoughts that produce uneasiness, apprehension, fear, disgust, or worry (obsessions), and repetitive ritualized behaviors that serve to reduce anxiety or unpleasant feelings (compulsions). Most children suffer from a combination of both. Without treatment, OCD can become a chronic and disabling disorder, with a population prevalence rate between 1-3% in children and adolescents .^1^

Description of the intervention

This systematic review focuses on internet cognitive behavioral therapy interventions for pediatric OCD. Cognitive behavioral therapy (CBT) is a psychotherapeutic approach that addresses dysfunctional emotions, maladaptive behaviors, and cognitive processes through a number of goal-oriented and systematic procedures .^2^ After years of research, CBT, in the form of exposure and response prevention (E/RP), is now considered the first line of treatment for pediatric OCD ^2^ ^3^ "Exposure" refers to confrontation with anxiety-provoking stimuli that trigger obsessions and/or compulsions. "Response Prevention" refers to not engaging in compulsive behavior after contact with the anxiety-provoking stimulus.

Background

Today, children grow up using smartphones, computers, and tablets on a daily base. These technologies, if integrated in a principled way, provide opportunities for enhancing the efficacy of CBT. New information technologies (IT) can make office-based CBT more accessible, more user-friendly, and more (cost-)effective. Also, adding new technologies may make treatment more appealing to children and adolescents, which may enhance treatment motivation, adherence, and treatment outcomes. Adding IT to CBT can be in the form of incorporating smartphones, webcams, tablets, and computers, and using applications such as e-mail, Short Message Service (SMS), blogs, social networks, instant messaging and podcasts.

Definition of Internet Cognitive Behavioral Therapy (iCBT)

In 2016 Andersson defines iCBT as therapist-guided and automated interventions that are delivered using the Internet and information-technology based on cognitive-behaviour therapy ^4^.

Why it is important to do this review

iCBT is a relatively new field, and technologies and interventions are developing fast. Although iCBT has increased substantially over the last ten years, no systematic review on iCBT for pediatric OCD has been previously published. We plan a review on the developments in this field that provides information on the existing interventions and the acceptability, feasibility and efficacy of the interventions. Given the existence of sufficient studies, this review will provide an overview of the strengths and weaknesses of different types of iCBT. This can help decide which hardware and software may be helpful and guide future integrations of technology into traditional CBT.

Objectives

The aim of this systematic review is to evaluate the acceptability, feasibility and efficacy of internet cognitive behavior therapy (iCBT) for children and adolescents with Obsessive Compulsive Disorder.

The goals of this study are to:

1. Assess the efficacy of iCBT.
2. Assess feasibility of iCBT.
3. Assess acceptability of iCBT.

Methods

Criteria for selecting studies for this review:

### 2.2.1. Inclusion criteria

Population

- Children and adolescents aged 4-18,
- Primary diagnosis of obsessive-compulsive disorder diagnosed by a psychologist or psychiatrist according to DSM or ICD criteria,
- All treatment settings,
- Any cultural background, ethnicity and sex.

Intervention

- CBT with internet technology components,
- No restrictions on therapist involvement or additional treatment.

Comparator

- Studies with and without comparators.

Outcome

- Acceptability, feasibility, or efficacy.

Study Design

- Randomized controlled trial, blind trial, non-blind trial, adaptive clinical trial, non-randomized trial, interrupted time series design, cohort study, case-control study, cross-sectional study published in English.

### 2.2.2. Exclusion criteria

Population

- Adults,
- Primary diagnosis of obsessive-compulsive disorder diagnosed by non-specialist (not psychologist or psychiatrist) or not according to DSM or ICD criteria.

Intervention

- Other than CBT or CBT without internet technology components.

Comparator

- Studies with and without comparators are accepted.

Outcome

- Not reporting on acceptability, feasibility and efficacy.

Study Design

- Qualitative study, commentary, correction, editorial letter (unless research letter reporting data).

*Three concepts*

- The condition: obsessive-compulsive disorder.
- Age group: 4-18 years old.
- Intervention: iCBT, different forms are possible.

*Outcome concepts*

Acceptability: Treatment acceptability refers to the degree to which an individual perceives a treatment protocol as appropriate, fair and reasonable for a given population or problem .^5^ Treatments deemed as unacceptable are less likely to be utilized regardless of their efficacy.^6^ Does it meet user (child, parent, therapist) expectations?

Acceptability is measured by:

- recommendation of iCBT to others with similar problems
- willing to accept an iCBT program in the future
- Parent and child treatment satisfaction
- Difficulties using iCBT: difficulties experienced by participants

Feasibility: Treatment feasibility refers whether iCBT can meet patients’ needs.^7^ Does is work in practice?

Feasibility is measured by:

- Rates of recruitment
- Rates of retention
- Number/percentage of completed sessions
- Homework compliance
- Implementation barriers
- Feedback from outside experts: clinicians, researchers
- Feedback from therapists
- Feedback from participants (children and parents)

Treatment efficacy refers to the capacity to improving healyh-outcomes. The efficacy of iCBT is the effect on OCD symptoms, overall functioning and family, academic and social functioning ^8^.

- The CY-BOCS is the golden standard for measuring efficacy in OCD treatment.

## Search methods for identification of studies

1. Electronic databases

We will search in the following databases for studies published in English:

- The Cochrane Central Register of Controlled Trials (CENTRAL)
- MEDLINE (Pubmed covered by MEDLINE)
- EMBASE
- PsycInfo
- LILACS
- CINAHL
- Scopus: largest abstract and citation database of peer-reviewed literature

The WHO Trials portal (ICTRP) and ClinicalTrials.gov will be searched to identify additional unpublished and/or ongoing studies.

The first publication that studied the efficacy of computer-based treatment of OCD was by Baer et al. (1987) and Baer et al. (1988). They provided case reports of a computerized intervention for adults with OCD.

Specific scientific journals form this to be scrutinized:

American Journal of Psychiatry

Cognitive Therapy and Research
Journal of Child Psychology and Psychiatry
British Journal of Psychiatry
Behavioral and Cognitive Psychotherapy
British Journal of Clinical Psychology

European Journal of Child and Adolescent Psychiatry

Journal of the American Academy of Child and Adolescent Psychiatry
Journal of Consulting and Clinical Psychology
Journal of Clinical Child and Adolescent Psychology
Journal of Abnormal Psychology
Journal of Abnormal Child Psychology
Journal of Behavior Therapy Experimental Psychiatry
Behavior Research and Therapy

2. Key authors and reference lists checking

The reference lists of all identified studies will be inspected for more published reports and citations of unpublished research. If necessary, the key authors will be inspected for additional studies.

3. Personal communication

If necessary, the lead author on included studies and other experts in the field will be approached to request details of any further published and/or unpublished studies.

4. Textbooks and conference abstracts

Conference abstracts available online for WCBCT, EABCT, ESCAP, ABCT, AACAP, IACAPAP, ICCAP, BABCP and ICOCS will be scrutinised for relevant references. Conference abstracts are an important source of grey literature.

*Data collection and analysis*

1. Selection of studies

(VB/LBE and SP) will conduct the initial searches by the four mentioned steps. Articles that don’t fulfil eligibility criteria as judged by titles and abstracts were rejected by the first review author (LBE). Two review authors (LBE and LW) will independently assess the titles and abstracts of the resulting lists of studies for relevance. We then will obtain full articles for potentially relevant abstracts.

2. Data extraction and management

A data extraction form will be designed by one of the review authors (VB). This form includes verification of study eligibility, general information about the study and information about the methodology, participants, intervention and outcome measures. Also, some participants lost or excluded at each stage of the trial will be included in the form. Review authors (VB/LBE and LW) independently assess the identified trials to determine eligibility using the data extraction spreadsheet, starting with a pilot test of eligibility criteria (12 articles) to refine and clarify the spreadsheet. In the case of disagreement concerning trial eligibility, we make the final decision by discussion and consensus, if necessary, with the involvement of another member of the review group (BW and NS).

Two review authors (LBE and LW) will independently extract data from the included studies using the data extraction spreadsheet. If a study does not provide complete information (for example, details of drop out or group means), we will contact the primary investigator by e-mail.

3. Assessment of risk of bias in included studies

(LBE and LW) assess the risk of bias in each included study using the Cochrane Collaboration’s ‘risk of bias’ tool .^9^ We assess the following six areas for risk of bias.

1. Sequence generation: was the allocation sequence of participants adequately randomized?
2. Allocation concealment: was the allocation sequence adequately concealed from participants as well as those involved in the enrolment and assignment of participants?
3. Blinding of outcome assessment: Where patients or assessing researchers aware of the condition as control or experimental?
4. Incomplete outcome data: were there incomplete data for the primary or secondary outcomes (e.g., due to attrition)? Were incomplete data adequately accounted for?
5. Selective reporting: was the study free of suggestions for selective reporting of outcomes (e.g., reporting of a subset of outcomes by the results)?

Two review authors (LBE and LW) will independently assess the risk of bias for each included study. Disagreements will be resolved by consensus and by discussion and with the involvement of another member of the review group (BW and NS). If further information about a particular trial is required to assess its risk of bias, we will attempt to contact the primary author of the relevant study. ‘Risk of bias' tables will be constructed that describes the information outlined above, as reported in each study. These tables will also include a judgement on the risk of bias, made by the review authors for each of the six areas, based on the following three categories: 1. low risk of bias, 2. high risk of bias, and 3. unclear or unknown risk of bias.

*Acknowledgements*

We would like to thank RKBU Midt Norge, Faculty of Medicine, NTNU for its support.

## Search strategy to use in the systematic review

We will search the following electronic bibliographic databases: MEDLINE, EMBASE, The Cochrane Library (Cochrane Database of Systematic Reviews, Cochrane Central Register of Controlled Trials (CENTRAL)), PsycINFO, CINAHL, LILACS, Scopus, ICTRP and ClinicalTrials.gov.

The search strategy will involve a combination of a thesaurus and free-text terms optimized to identify English-language literature that deals with iCBT in children and adolescents with obsessive-compulsive disorder. Since the first publication on computerized interventions for the obsessive-compulsive disorder was published by Baer et al. 1987, the search will be limited to literature published in 1987 or later. To identify potentially relevant studies not discovered in the searched databases, reference lists of eligible studies and review articles, in addition to abstracts from relevant journals, will also be screened. The corresponding author include studies and other experts in the field will contact to obtained details on published or unpublished studies. The search will be updated just prior to the final analyses to ensure that new and potentially relevant publications are included.

**Specification of the search strategy (suitable for inclusion in supplementary information or an appendix)**

**Medline via OVID**

Ovid MEDLINE(R) Epub Ahead of Print, In-Process & Other Non-Indexed Citations, Ovid MEDLINE(R) Daily and Ovid MEDLINE(R) <1946 to Present>

| **#** | **Searches** |  |  |  |
| --- | --- | --- | --- | --- |
| 1 | exp Obsessive-Compulsive Disorder/ |  |  |  |
| 2 | ocd.ti,ab,kw. |  |  |  |
| 3 | obsess*.ti,ab,kw. |  |  |  |
| 4 | compulsi*.ti,ab,kw. |  |  |  |
| 5 | or/1-4 |  |  |  |
| 6 | exp Telemedicine/ |  |  |  |
| 7 | exp Electronic Mail/ |  |  |  |
| 8 | exp Telephone/ |  |  |  |
| 9 | exp Videoconferencing/ |  |  |  |
| 10 | exp Internet/ |  |  |  |
| 11 | exp Computers/ |  |  |  |
| 12 | exp Software/ |  |  |  |
| 13 | exp Decision Making, Computer-Assisted/ |  |  |  |
| 14 | smartphone*.ti,ab,kw. |  |  |  |
| 15 | telephone*.ti,ab,kw. |  |  |  |
| 16 | computer*.ti,ab,kw. |  |  |  |
| 17 | distance*.ti,ab,kw. |  |  |  |
| 18 | remote.ti,ab,kw. |  |  |  |
| 19 | internet*.ti,ab,kw. |  |  |  |
| 20 | telepsychiatry.ti,ab,kw. |  |  |  |
| 21 | telepsychology.ti,ab,kw. |  |  |  |
| 22 | telemental.ti,ab,kw. |  |  |  |
| 23 | telehealth.ti,ab,kw. |  |  |  |
| 24 | teletherapy.ti,ab,kw. |  |  |  |
| 25 | cybercounseling.ti,ab,kw. |  |  |  |
| 26 | (web adj2 based).ti,ab,kw. |  |  |  |
| 27 | "web based".ti,ab,kw. |  |  |  |
| 28 | phone*.ti,ab,kw. |  |  |  |
| 29 | mobile*.ti,ab,kw. |  |  |  |
| 30 | "e mail*".ti,ab,kw. |  |  |  |
| 31 | email*.ti,ab,kw. |  |  |  |
| 32 | "electronic mail*".ti,ab,kw. |  |  |  |
| 33 | online.ti,ab,kw. |  |  |  |
| 34 | "on line".ti,ab,kw. |  |  |  |
| 35 | videoconferenc*.ti,ab,kw. |  |  |  |
| 36 | "video conferenc*".ti,ab,kw. |  |  |  |
| 37 | (chat adj2 room*).ti,ab,kw. |  |  |  |
| 38 | "chat room*".ti,ab,kw. |  |  |  |
| 39 | (instant adj2 messaging).ti,ab,kw. |  |  |  |
| 40 | "instant messaging".ti,ab,kw. |  |  |  |
| 41 | iCBT.ti,ab,kw. |  |  |  |
| 42 | iCBTs.ti,ab,kw. |  |  |  |
| 43 | "social media?".ti,ab,kw. |  |  |  |
| 44 | or/6-43 |  |  |  |
| 45 | exp Child/ |  |  |  |
| 46 | exp Adolescent/ |  |  |  |
| 47 | child*.ti,ab,kw. |  |  |  |
| 48 | teen*.ti,ab,kw. |  |  |  |
| 49 | (young adj2 people*).ti,ab,kw. |  |  |  |
| 50 | "young people*".ti,ab,kw. |  |  |  |
| 51 | (young adj2 person*).ti,ab,kw. |  |  |  |
| 52 | "young person*".ti,ab,kw. |  |  |  |
| 53 | girl*.ti,ab,kw. |  |  |  |
| 54 | boy*.ti,ab,kw. |  |  |  |
| 55 | minor*.ti,ab,kw. |  |  |  |
| 56 | kid*.ti,ab,kw. |  |  |  |
| 57 | juvenile*.ti,ab,kw. |  |  |  |
| 58 | youth.ti,ab,kw. |  |  |  |
| 59 | adolesc*.ti,ab,kw. |  |  |  |
| 60 | p?ediatri*.ti,ab,kw. |  |  |  |
| 61 | or/45-60 |  |  |  |
| 62 | 5 and 44 and 61 |  |  |  |
| 63 | limit 62 to yr="1987 -Current" |  |  |  |

**Cochrane Library**

ID Search Hits

#1 MeSH descriptor: [Obsessive-Compulsive Disorder] explode all trees

#2 "ocd":ti,ab,kw (Word variations have been searched)

#3 obsess*:ti,ab,kw (Word variations have been searched)

#4 compulsi*:ti,ab,kw (Word variations have been searched)

#5 #1 or #2 or #3 or #4

#6 MeSH descriptor: [Telemedicine] explode all trees

#7 MeSH descriptor: [Electronic Mail] explode all trees

#8 MeSH descriptor: [Telephone] explode all trees

#9 MeSH descriptor: [Videoconferencing] explode all trees

#10 MeSH descriptor: [Internet] explode all trees

#11 MeSH descriptor: [Software] explode all trees

#12 MeSH descriptor: [Computers] explode all trees

#13 MeSH descriptor: [Decision Making, Computer-Assisted] explode all trees

#14 smartphone*:ti,ab,kw (Word variations have been searched)

#15 telephone*:ti,ab,kw (Word variations have been searched)

#16 computer*:ti,ab,kw (Word variations have been searched)

#17 distance*:ti,ab,kw (Word variations have been searched)

#18 remote:ti,ab,kw (Word variations have been searched)

#19 internet*:ti,ab,kw (Word variations have been searched)

#20 telepsychiatry:ti,ab,kw (Word variations have been searched)

#21 telepsychology:ti,ab,kw (Word variations have been searched)

#22 telemental:ti,ab,kw (Word variations have been searched)

#23 telehealth:ti,ab,kw (Word variations have been searched)

#24 teletherapy:ti,ab,kw (Word variations have been searched)

#25 cybercounseling:ti,ab,kw (Word variations have been searched)

#26 (web near/2 based):ti,ab,kw (Word variations have been searched)

#27 phone*:ti,ab,kw (Word variations have been searched)

#28 mobile*:ti,ab,kw (Word variations have been searched)

#29 "e mail*":ti,ab,kw (Word variations have been searched)

#30 email*:ti,ab,kw (Word variations have been searched)

#31 "electronic mail*":ti,ab,kw (Word variations have been searched)

#32 online:ti,ab,kw (Word variations have been searched)

#33 "on line":ti,ab,kw (Word variations have been searched)

#34 videoconferenc*:ti,ab,kw (Word variations have been searched)

#35 "video conferenc*":ti,ab,kw (Word variations have been searched)

#36 (chat near/2 room*):ti,ab,kw (Word variations have been searched)

#37 (instant near/2 messaging):ti,ab,kw (Word variations have been searched)

#38 iCBT:ti,ab,kw (Word variations have been searched)

#39 iCBTs:ti,ab,kw (Word variations have been searched)

#40 "social media*":ti,ab,kw (Word variations have been searched)

#41 #6 or #7 or #8 or #9 or #10 or #11 or #12 or #13 or #14 or #15 or #16 or #17 or #18 or #19 or #20 or #21 or #22 or #23 or #24 or #25 or #26 or #27 or #28 or #29 or #30 or #31 or #32 or #33 or #34 or #35 or #36 or #37 or #38 or #39 or #40

#42 MeSH descriptor: [Child] explode all trees

#43 MeSH descriptor: [Adolescent] explode all trees

#44 child*:ti,ab,kw (Word variations have been searched)

#45 teen*:ti,ab,kw (Word variations have been searched)

#46 (young near/2 people*):ti,ab,kw (Word variations have been searched)

#47 (young near/2 person*):ti,ab,kw (Word variations have been searched)

#48 girl*:ti,ab,kw (Word variations have been searched)

#49 boy*:ti,ab,kw (Word variations have been searched)

#50 minor*:ti,ab,kw (Word variations have been searched)

#51 kid*:ti,ab,kw (Word variations have been searched)

#52 juvenile*:ti,ab,kw (Word variations have been searched)

#53 youth:ti,ab,kw (Word variations have been searched)

#54 adolesc*:ti,ab,kw (Word variations have been searched)

#55 pediatri*:ti,ab,kw (Word variations have been searched)

#56 paediatri*:ti,ab,kw (Word variations have been searched)

#57 #42 or #43 or #44 or #45 or #46 or #47 or #48 or #49 or #50 or #51 or #52 or #53 or #54 or #55 or #56

#58 #5 and #41 and #57

**EMBASE via OVID**

Embase <1974 to 2017 June 26>

| **#** | **Searches** |  |  |  |
| --- | --- | --- | --- | --- |
| 1 | exp Obsessive-Compulsive Disorder/ |  |  |  |
| 2 | ocd.ti,ab,kw. |  |  |  |
| 3 | obsess*.ti,ab,kw. |  |  |  |
| 4 | compulsi*.ti,ab,kw. |  |  |  |
| 5 | or/1-4 |  |  |  |
| 6 | exp Telehealth/ |  |  |  |
| 7 | exp Mass communication/ |  |  |  |
| 8 | exp Software/ |  |  |  |
| 9 | exp Computer/ |  |  |  |
| 10 | exp Computer-assisted therapy/ |  |  |  |
| 11 | smartphone*.ti,ab,kw. |  |  |  |
| 12 | telephone*.ti,ab,kw. |  |  |  |
| 13 | computer*.ti,ab,kw. |  |  |  |
| 14 | distance*.ti,ab,kw. |  |  |  |
| 15 | remote.ti,ab,kw. |  |  |  |
| 16 | internet*.ti,ab,kw. |  |  |  |
| 17 | telepsychiatry.ti,ab,kw. |  |  |  |
| 18 | telepsychology.ti,ab,kw. |  |  |  |
| 19 | telemental.ti,ab,kw. |  |  |  |
| 20 | telehealth.ti,ab,kw. |  |  |  |
| 21 | teletherapy.ti,ab,kw. |  |  |  |
| 22 | cybercounseling.ti,ab,kw. |  |  |  |
| 23 | (web adj2 based).ti,ab,kw. |  |  |  |
| 24 | "web based".ti,ab,kw. |  |  |  |
| 25 | phone*.ti,ab,kw. |  |  |  |
| 26 | mobile*.ti,ab,kw. |  |  |  |
| 27 | "e mail*".ti,ab,kw. |  |  |  |
| 28 | email*.ti,ab,kw. |  |  |  |
| 29 | "electronic mail*".ti,ab,kw. |  |  |  |
| 30 | online.ti,ab,kw. |  |  |  |
| 31 | "on line".ti,ab,kw. |  |  |  |
| 32 | videoconferenc*.ti,ab,kw. |  |  |  |
| 33 | "video conferenc*".ti,ab,kw. |  |  |  |
| 34 | (chat adj2 room*).ti,ab,kw. |  |  |  |
| 35 | "chat room*".ti,ab,kw. |  |  |  |
| 36 | (instant adj2 messaging).ti,ab,kw. |  |  |  |
| 37 | "instant messaging".ti,ab,kw. |  |  |  |
| 38 | iCBT.ti,ab,kw. |  |  |  |
| 39 | iCBTs.ti,ab,kw. |  |  |  |
| 40 | "social media?".ti,ab,kw. |  |  |  |
| 41 | or/6-40 |  |  |  |
| 42 | exp Child/ |  |  |  |
| 43 | exp Adolescent/ |  |  |  |
| 44 | child*.ti,ab,kw. |  |  |  |
| 45 | teen*.ti,ab,kw. |  |  |  |
| 46 | (young adj2 people*).ti,ab,kw. |  |  |  |
| 47 | "young people*".ti,ab,kw. |  |  |  |
| 48 | (young adj2 person*).ti,ab,kw. |  |  |  |
| 49 | "young person*".ti,ab,kw. |  |  |  |
| 50 | girl*.ti,ab,kw. |  |  |  |
| 51 | boy*.ti,ab,kw. |  |  |  |
| 52 | minor*.ti,ab,kw. |  |  |  |
| 53 | kid*.ti,ab,kw. |  |  |  |
| 54 | juvenile*.ti,ab,kw. |  |  |  |
| 55 | youth.ti,ab,kw. |  |  |  |
| 56 | adolesc*.ti,ab,kw. |  |  |  |
| 57 | p?ediatri*.ti,ab,kw. |  |  |  |
| 58 | or/42-57 |  |  |  |
| 59 | 5 and 41 and 58 |  |  |  |
| 60 | limit 59 to yr="1987 -Current" |  |  |  |

**PsycINFO via OVID**

PsycINFO <1987 to June Week 3 2017>

| **#** | **Searches** |  |  |  |
| --- | --- | --- | --- | --- |
| 1 | exp Obsessive Compulsive Disorder/ |  |  |  |
| 2 | ocd.ti,ab,id. |  |  |  |
| 3 | obsess*.ti,ab,id. |  |  |  |
| 4 | compulsi*.ti,ab,id. |  |  |  |
| 5 | or/1-4 |  |  |  |
| 6 | exp Telemedicine/ |  |  |  |
| 7 | exp Computers/ |  |  |  |
| 8 | exp Computer Software/ |  |  |  |
| 9 | exp Computer Applications/ |  |  |  |
| 10 | exp Computer conferencing/ |  |  |  |
| 11 | exp Internet/ |  |  |  |
| 12 | exp Telecommunications Media/ |  |  |  |
| 13 | exp Electronic Communication/ |  |  |  |
| 14 | exp Internet Usage/ |  |  |  |
| 15 | exp Online Therapy/ |  |  |  |
| 16 | smartphone*.ti,ab,id. |  |  |  |
| 17 | telephone*.ti,ab,id. |  |  |  |
| 18 | computer*.ti,ab,id. |  |  |  |
| 19 | distance*.ti,ab,id. |  |  |  |
| 20 | remote.ti,ab,id. |  |  |  |
| 21 | internet*.ti,ab,id. |  |  |  |
| 22 | telepsychiatry.ti,ab,id. |  |  |  |
| 23 | telepsychology.ti,ab,id. |  |  |  |
| 24 | telemental.ti,ab,id. |  |  |  |
| 25 | telehealth.ti,ab,id. |  |  |  |
| 26 | teletherapy.ti,ab,id. |  |  |  |
| 27 | cybercounseling.ti,ab,id. |  |  |  |
| 28 | (web adj2 based).ti,ab,id. |  |  |  |
| 29 | "web based".ti,ab,id. |  |  |  |
| 30 | phone*.ti,ab,id. |  |  |  |
| 31 | mobile*.ti,ab,id. |  |  |  |
| 32 | "e mail*".ti,ab,id. |  |  |  |
| 33 | email*.ti,ab,id. |  |  |  |
| 34 | "electronic mail*".ti,ab,id. |  |  |  |
| 35 | online.ti,ab,id. |  |  |  |
| 36 | "on line".ti,ab,id. |  |  |  |
| 37 | videoconferenc*.ti,ab,id. |  |  |  |
| 38 | "video conferenc*".ti,ab,id. |  |  |  |
| 39 | (chat adj2 room*).ti,ab,id. |  |  |  |
| 40 | "chat room*".ti,ab,id. |  |  |  |
| 41 | (instant adj2 messaging).ti,ab,id. |  |  |  |
| 42 | "instant messaging".ti,ab,id. |  |  |  |
| 43 | iCBT.ti,ab,id. |  |  |  |
| 44 | iCBTs.ti,ab,id. |  |  |  |
| 45 | "social media?".ti,ab,id. |  |  |  |
| 46 | or/6-45 |  |  |  |
| 47 | child*.ti,ab,id. |  |  |  |
| 48 | teen*.ti,ab,id. |  |  |  |
| 49 | (young adj2 people*).ti,ab,id. |  |  |  |
| 50 | "young people*".ti,ab,id. |  |  |  |
| 51 | (young adj2 person*).ti,ab,id. |  |  |  |
| 52 | "young person*".ti,ab,id. |  |  |  |
| 53 | girl*.ti,ab,id. |  |  |  |
| 54 | boy*.ti,ab,id. |  |  |  |
| 55 | minor*.ti,ab,id. |  |  |  |
| 56 | kid*.ti,ab,id. |  |  |  |
| 57 | juvenile*.ti,ab,id. |  |  |  |
| 58 | youth.ti,ab,id. |  |  |  |
| 59 | adolesc*.ti,ab,id. |  |  |  |
| 60 | p?ediatri*.ti,ab,id. |  |  |  |
| 61 | or/47-60 |  |  |  |
| 62 | 5 and 46 |  |  |  |
| 63 | limit 62 to (childhood <birth to 12 years> or adolescence <13 to 17 years>) |  |  |  |
| 64 | 5 and 46 and 61 |  |  |  |
| 65 | 63 or 64 |  |  |  |
| 66 | limit 65 to yr="1987 -Current" |  |  |  |

**LILACS - Latin American and Caribbean Health Sciences**

1. MH Obsessive-Compulsive Disorder OR MH ocd OR TW obsess$ OR Tw compulsi$
2. MH Telemedicine OR MH Electronic Mail OR MH Telephone OR MH Videoconferencing OR MH Internet OR MH Computers OR MH Software OR MH Decision Making, Computer-Assisted OR TW smartphone$ OR TW telephone$ OR TW computer$ OR TW distance$ OR TW remote OR TW internet$ OR TW telepsychiatry OR TW telepsychology OR TW telemental OR TW telehealth OR TW teletherapy OR TW cybercounseling OR TW web AND TW based OR TW phone$ OR TW mobile$ OR TW e AND TW mail$ OR TW email$ OR TW electronic TW mail$ OR TW online OR TW on AND TW line OR TW videoconferenc$ OR TW video AND TW conferenc$ OR TW chat AND TW room$ OR TW instant AND TW messaging OR TW iCBT OR TW iCBTs OR TW social AND TW media$
3. MH Child OR MH Adolescent OR TW child$ OR TW teen$ OR TW young AND TW people$ OR TW young AND TW person$ OR TW girl$ OR TW boy$ OR TW minor$ OR TW kid$ OR TW juvenile$ OR TW youth OR TW adolesc$ OR TW pediatri$ OR TW paediatri$
4. #1 AND #2 AND #3

CINAHL

S53 S5 AND S35 AND S51 Limiters - Published Date: 19870101-20171231

Search modes - Boolean/Phrase

S52 S5 AND S35 AND S51

S51 S36 OR S37 OR S38 OR S39 OR S40 OR S41 OR S42 OR S43 OR S44 OR S45 OR S46 OR S47 OR S48 OR S49 OR S50

S50 TI p#ediatri* OR AB p#ediatri*

S49 TI adolesc* OR AB adolesc*

S48 TI youth OR AB youth

S47 TI juvenile* OR AB juvenile*

S46 TI teen* OR AB teen*

S45 TI kid* OR AB kid*

S44 TI minor* OR AB minor*

S43 TI boy* OR AB boy*

S42 TI girl* OR AB girl*

S41 TI (young N2 person*) OR AB (young N2 person*)

S40 TI (young N2 people*) OR AB (young N2 people*)

S39 TI teen* OR AB teen*

S38 TI child* OR AB child*

S37 (MH "Adolescence+")

S36 (MH "Child+")

S35 S6 OR S7 OR S8 OR S9 OR S10 OR S11 OR S12 OR S13 OR S14 OR S15 OR S16 OR S17 OR S18 OR S19 OR S20 OR S21 OR S22 OR S23 OR S24 OR S25 OR S26 OR S27 OR S28 OR S29 OR S30 OR S31 OR S32 OR S33 OR S34

S34 TI "social media?" OR AB "social media?"

S33 TI iCBTs OR AB iCBTs

S32 TI iCBT OR AB iCBT

S31 TI (instant Nj2 messaging) OR AB (instant N2 messaging)

S30 TI (chat N2 room*) OR AB (chat N2 room*)

S29 TI "video conferenc*" OR AB "video conferenc*"

S28 TI videoconferenc* OR AB videoconferenc*

S27 TI "on line" OR AB "on line"

S26 TI online OR AB online

S25 TI "electronic mail*" OR AB "electronic mail*"

S24 TI email* OR AB email*

S23 TI "e mail* OR AB "e mail*

S22 TI mobile* OR AB mobile*

S21 TI phone* OR AB phone*

S20 TI (web N2 based) OR AB (web N2 based)

S19 TI cybercounseling OR AB cybercounseling

S18 TI teletherapy OR AB teletherapy

S17 TI telehealth OR AB telehealth

S16 TI telemental OR AB telemental

S15 TI telepsychology OR AB telepsychology

S14 TI telepsychiatry OR AB telepsychiatry

S13 TI internet* OR AB internet*

S12 TI remote OR AB remote

S11 TI distance* OR AB distance*

S10 TI computer* OR AB computer*

S9 TI telephone* OR AB telephone*

S8 TI smartphone* OR AB smartphone*

S7 (MH "Telecommunications+")

S6 (MH "Computers and Computerization+")

S5 S1 OR S2 OR S3 OR S4

S4 TI compulsi* OR AB compulsi*

S3 TI obsess* OR AB obsess*

S2 TI ocd OR AB ocd

S1 (MH "Obsessive-Compulsive Disorder+")

**Scopus**

( ( TITLE-ABS-KEY ( ocd ) )  OR  ( TITLE-ABS-KEY ( obsess* ) )  OR  ( TITLE-ABS-KEY ( compulsi* ) ) )  AND  ( ( TITLE-ABS-KEY ( smartphone* ) )  OR  ( TITLE-ABS-KEY ( telephone* ) )  OR  ( TITLE-ABS-KEY ( computer* ) )  OR  ( TITLE-ABS-KEY ( distance* ) )  OR  ( TITLE-ABS-KEY ( remote ) )  OR  ( TITLE-ABS-KEY ( internet* ) )  OR  ( TITLE-ABS-KEY ( telepsychiatry ) )  OR  ( TITLE-ABS-KEY ( telepsychology ) )  OR  ( TITLE-ABS-KEY ( telemental ) )  OR  ( TITLE-ABS-KEY ( telehealth ) )  OR  ( TITLE-ABS-KEY ( teletherapy ) )  OR  ( TITLE-ABS-KEY ( cybercounseling ) )  OR  ( TITLE-ABS-KEY ( ( web )  PRE/2  ( based ) ) )  OR  ( TITLE-ABS-KEY ( phone* ) )  OR  ( TITLE-ABS-KEY ( phone* ) )  OR  ( TITLE-ABS-KEY ( mobile* ) )  OR  ( TITLE-ABS-KEY ( "e mail*" ) )  OR  ( TITLE-ABS-KEY ( email* ) )  OR  ( TITLE-ABS-KEY ( "electronic mail*" ) )  OR  ( TITLE-ABS-KEY ( online ) )  OR  ( TITLE-ABS-KEY ( "on line" ) )  OR  ( TITLE-ABS-KEY ( videoconferenc* ) )  OR  ( TITLE-ABS-KEY ( "video conferenc*" ) )  OR  ( TITLE-ABS-KEY ( ( chat )  PRE/2  ( room* ) ) )  OR  ( TITLE-ABS-KEY ( ( instant )  PRE/2  ( messaging ) ) )  OR  ( TITLE-ABS-KEY ( icbt ) )  OR  ( TITLE-ABS-KEY ( icbts ) )  OR  ( TITLE-ABS-KEY ( "social media" ) )  OR  ( TITLE-ABS-KEY ( "social medias" ) ) )  AND  ( ( TITLE-ABS-KEY ( child* ) )  OR  ( TITLE-ABS-KEY ( teen* ) )  OR  ( TITLE-ABS-KEY ( ( young )  W/2  ( people* ) ) )  ( TITLE-ABS-KEY ( ( young )  W/2  ( person* ) ) )  OR  ( TITLE-ABS-KEY ( girl* ) )  OR  ( TITLE-ABS-KEY ( boy* ) )  OR  ( TITLE-ABS-KEY ( minor* ) )  OR  ( TITLE-ABS-KEY ( kid* ) )  OR  ( TITLE-ABS-KEY ( juvenile* ) )  OR  ( TITLE-ABS-KEY ( youth ) )  OR  ( TITLE-ABS-KEY ( adolesc* ) )  OR  ( TITLE-ABS-KEY ( pediatri* ) )  OR  ( TITLE-ABS-KEY ( paediatri* ) ) )  AND  ( LIMIT-TO ( PUBYEAR ,  2017 )  OR  LIMIT-TO ( PUBYEAR ,  2016 )  OR  LIMIT-TO ( PUBYEAR ,  2015 )  OR  LIMIT-TO ( PUBYEAR ,  2014 )  OR  LIMIT-TO ( PUBYEAR ,  2013 )  OR  LIMIT-TO ( PUBYEAR ,  2012 )  OR  LIMIT-TO ( PUBYEAR ,  2011 )  OR  LIMIT-TO ( PUBYEAR ,  2010 )  OR  LIMIT-TO ( PUBYEAR ,  2009 )  OR  LIMIT-TO ( PUBYEAR ,  2008 )  OR  LIMIT-TO ( PUBYEAR ,  2007 )  OR  LIMIT-TO ( PUBYEAR ,  2006 )  OR  LIMIT-TO ( PUBYEAR ,  2005 )  OR  LIMIT-TO ( PUBYEAR ,  2004 )  OR  LIMIT-TO ( PUBYEAR ,  2003 )  OR  LIMIT-TO ( PUBYEAR ,  2002 )  OR  LIMIT-TO ( PUBYEAR ,  2001 )  OR  LIMIT-TO ( PUBYEAR ,  2000 )  OR  LIMIT-TO ( PUBYEAR ,  1999 )  OR  LIMIT-TO ( PUBYEAR ,  1998 )  OR  LIMIT-TO ( PUBYEAR ,  1997 )  OR  LIMIT-TO ( PUBYEAR ,  1996 )  OR  LIMIT-TO ( PUBYEAR ,  1994 )  OR  LIMIT-TO ( PUBYEAR ,  1993 )  OR  LIMIT-TO ( PUBYEAR ,  1992 )  OR  LIMIT-TO ( PUBYEAR ,  1991 )  OR  LIMIT-TO ( PUBYEAR ,  1989 )  OR  LIMIT-TO ( PUBYEAR ,  1988 )  OR  LIMIT-TO ( PUBYEAR ,  1987 ) )

**WHO International Clinical Trials Registry Platform**

Title: smartphone* OR telephone* OR computer* OR distance OR remote OR internet* OR telepsychiatry OR telepsychology OR telemental OR telehealth OR teletherapy OR cybercounseling OR "web based" OR phone* OR mobile* OR "e mail" OR email OR "electronic mail" OR oneline OR "on line" OR videoconference OR "video conference" OR "chat room" OR "chat rooms" OR "instant messaging" OR ICBT OR ICBTs OR "social media"

AND

Condition: ocd OR obsess* OR compulsi*

**ClinicalTrial.gov**

Condition/Disease: ocd OR obsess* OR compulsi*

Other Terms: smartphone* OR telephone* OR computer* OR distance OR remote OR internet* OR telepsychiatry OR telepsychology OR telemental OR telehealth OR teletherapy OR cybercounseling OR "web based" OR phone* OR mobile* OR "e mail" OR email OR "electronic mail" OR oneline OR "on line" OR videoconference OR "video conference" OR "chat room" OR "chat rooms" OR "instant messaging" OR ICBT OR ICBTs OR "social media"

Librarian: Sindre A. Pedersen, Ph.D, Førstebibliotekar, NTNU, Bibliotek for Medisin og Helse

## Contributions of authors

Lucía Babiano-Espinosa: *“The acquisition, analysis, and interpretation of data for the work (assessing independently the identified trials to determine eligibility, risk of bias assessment). Drafting the work. Final approval of the version to be published. Agreement to be accountable for all aspects of the work in ensuring that questions related to the accuracy or integrity of any part of the work are appropriately investigated and resolved.”* ^10^

Lidewij Wolters: *“Conception and design of the work, the acquisition, analysis, and interpretation of data for the work (assessing independently the identified trials to determine eligibility and risk of bias assessment), revising the work critically for important intellectual content, final approval of the version to be published, agreement to be accountable for all aspects of the work in ensuring that questions related to the accuracy or integrity of any part of the work are appropriately investigated and resolved.” ^10^*

Bernhard Weidle: *“Design of the work, revising the work critically for important intellectual content, final approval of the version to be published, agreement to be accountable for all aspects of the work in ensuring that questions related to the accuracy or integrity of any part of the work are appropriately investigated and resolved.” ^10^*

Vivian op de Beek: *“Conception and design of the work (protocol drafting), the acquisition, analysis, interpretation of data for the work (preliminar searches), revising the work critically for important intellectual content, final approval of the version to be published, agreement to be accountable for all aspects of the work in ensuring that questions related to the accuracy and integrity of any part of the work are appropriately investigated and resolved.” ^10^*

Sindre Pedersen: *“The acquisition and analysis (database searches), revising the work critically for important intellectual content, final approval of the version to be published, agreement to be accountable for all aspects of the work in ensuring that questions related to the accuracy or integrity of any part of the work are appropriately investigated and resolved.” ^10^*

Scott Compton: *“Design of the work, revising the work critically for important intellectual content, final approval of the version to be published, agreement to be accountable for all aspects of the work in ensuring that questions related to the accuracy or integrity of any part of the work are appropriately investigated and resolved.” ^10^*

Norbert Skokauskas: *“Conception and design of the work, the acquisition, analysis, and interpretation of data for the work (assessing independently the identified trials to determine eligibility and risk of bias assessment), revising the work critically for important intellectual content, final approval of the version to be published, agreement to be accountable for all aspects of the work in ensuring that questions related to the accuracy and integrity of any part of the work are appropriately investigated and resolved.” ^10^*

## Declarations of interest

Lucía Babiano-Espinosa, Lidewij Wolters, Bernhard Weidle, Vivian op de Beek, Sindre Pedersen, Norbert Skokauskas have no known conflicts of interest.

## Sources of support

*Internal sources*

- RKBU Midt Norge, Faculty of Medicine, NTNU, Trondheim

*External sources*

- Funding by Samarbeidsorganet mellom Helse Midt-Norge RHF and NTNU.

## References

**1.** Moore PS, Mariaskin A, March J, Franklin ME, Storch E, Geffken G. Obsessive-compulsive disorder in children and adolescents: diagnosis, comorbidity, and developmental factors. *Handbook of child and adolescent obsessive-compulsive disorder.* 2007:17-45.

**2.** AACAP. Practice parameter for the assessment and treatment of children and adolescents with obsessive-compulsive disorder. *J Am Acad Child Adolesc Psychiatry.* 2012;51((1)):98-113.

**3.** Excellence. NIfHaC. Obsessive-compulsive disorder (OCD) and body dysmorphic disorder (BDD). *National Institute for Health and Clinical Excellence.* 2005.

**4.** Andersson G, Carlbring P, Lindefors N. History and current status of ICBT. *Guided internet-based treatments in psychiatry*: Springer; 2016:1-16.

**5.** Tarrier N, Liversidge T, Gregg L. The acceptability and preference for the psychological treatment of PTSD. *Behaviour Research and Therapy.* 2006;44(11):1643-1656.

**6.** Miller DN, DuPaul GJ, Lutz JG. School-based psychosocial interventions for childhood depression: Acceptability of treatments among school psychologists. *School Psychology Quarterly.* 2002;17(1):78.

**7.** Myers KM, Valentine JM, Melzer SM. Feasibility, acceptability, and sustainability of telepsychiatry for children and adolescents. *Psychiatric Services.* 2007;58(11):1493-1496.

**8.** McGuire JF, Piacentini J, Lewin AB, Brennan EA, Murphy TK, Storch EA. A meta‐analysis of cognitive behavior therapy and medication for child obsessive–compulsive disorder: Moderators of treatment efficacy, response, and remission. *Depress. Anxiety.* 2015;32(8):580-593.

**9.** Higgins JP, Altman DG, Gøtzsche PC, et al. The Cochrane Collaboration’s tool for assessing risk of bias in randomised trials. *BMJ,.* 2011;343:d5928.

**10.** JAACAP. Guide for Authors. *The Journal of the American Academy of Child and Adolescent Psychiatry (JAACAP).* 2018.
