# Supplementary material for: Acceptability, feasibility, and efficacy of Internet cognitive behavioral therapy (iCBT) for pediatric obsessive-compulsive disorder: a systematic review
Source: Syst Rev. 2019 Nov 20;8:284. doi: 10.1186/s13643-019-1166-6 (PMC6864940; doi:10.1186/s13643-019-1166-6)
Supplement: Supplementary file 3 — Additional file 3. Overview of assessment instruments. [file 13643_2019_1166_MOESM3_ESM.docx]

| Additional File 3. Overview of assessment instruments | | | | | |
| --- | --- | --- | --- | --- | --- |
| Abbreviation | Full Name | Study | Purpose | Respondent | Interpretation |
| ADIS (CSR) | The Anxiety Disorders Interview Schedule | Comer et al. 2014, Comer et al. 2017 | Assesses child psychopathology in accordance to DSM–IV | Self-Assessment & Parents | Scores 0 – 8, with a score of 4 or higher indicating a clinically significant diagnosis |
| CDI | Children’s Depression Inventory | Farrell et al. 2016 | Rates severity of symptoms related to depression or dysthymic disorder in a pediatric sample | Self-Assessment | Scores range between 0-54  19–20 is generally accepted as depression  36 or higher relatively severe depression |
| CGAS | The Children's Global Assessment Scale | Lenhard et al. 2017 and Lenhard et al. 2014 | Rates the general functioning of patients under the age of 18 | Clinician | 10–1 Needs constant supervision  20–11 Needs considerable supervision  30–21 Unable to function in almost all areas  40–31 Major impairment of functioning in several areas  50–41 Moderate degree of interference in functioning in most social areas or severe impairment of functioning in one area  60–51 Variable functioning with sporadic difficulties or symptoms in several but not all social areas  70–61 Some difficulty in a single area but generally functioning well  80–71 No more than slight impairments in functioning at home, at school, or with peers; some disturbance of behavior or emotional distress may be present in response to life stresses  90–81 Good functioning in all areas  100–91 Superior functioning in all areas |
| CGI | The Clinical Global Impression | All | Measures symptom severity and treatment response in patients with [mental disorders](https://en.wikipedia.org/wiki/Mental_disorders) | Clinician | Severity Scale:  1 -Normal, not at all ill  2 -Borderline mentally ill  3- Mildly ill  4- Moderately ill  5- Markedly ill  6- Severely ill  7- Among the most extremely ill patients  Improvement Scale:  1 -Very much improved  2 -Much improved  3 -Minimally improved  4 -No change  5 -Minimally worse  6 -Much worse  7 -Very much worse |
| ChOCI-R  (symptom/impairment  Parent) (symptom/impairment  Child) | Children’s Obsessional Compulsive Inventory-Revised | Lenhard et al. 2017 and Lenhard et al. 2014 | Assesses the content and impairment of compulsions and obsessions in children and adolescents aged 7-17 years | Self-Assessment & Parents | 32-item, two-part measure assessing the content and severity of compulsions and obsessions in children and adolescents aged 7-17 years.  It provides a raw score for compulsion and obsession, and also a raw score for total impairment from 0 to 48 and total symptoms from 0 to 40. Total impairment score of >17 |
| COIS  Child/Parent | Child OCD Impact Scale | Storch et al. 2011 and Lenhard et al. 2014 | Assesses the impact of OCD symptoms on psychosocial functioning in children and adolescents | Self-Assessment & Parents | 4-point Likert-scale:  0 -Not at all  1-Just a Little  2-Pretty Much  3-Very Much  Maximum possible score of 99, with higher scores indicating more impairment. |
| CSQ-8 | The Client Satisfaction Questionnaire | Comer et al. 2014 and Comer et al. 2017 | Assesses client satisfaction with health, human services, governmental and public benefit programs and services | Self-Assessment & Parents | Scores range from 8 to 32, with higher values indicating higher satisfaction. |
| CY-BOCS  Child/Parent | The Children’s Yale-Brown Obsessive-Compulsive Scale | All | Rates the severity of  OCD symptoms | Clinician | 0–7 subclinical  8–15 mild  16–23 moderate  24–31 severe  32–40 extreme |
| EWSAS  Child / Parent | Education, Work and Social Adjustment Scale | Lenhard et al. 2017 | This assessment is an adaptation of the Work and Social Adjustment Scale for children and adolescents and rates the degree of impairment of the patient | Self-Assessment | Tests 5 different areas of functioning (school, everyday situations, social activities, leisure time, family and relationships). Higher ratings indicating more impairment, scores range from 0 to 40. |
| FAS | Family Accommodation Scale | Comer et al. 2014 and Comer et al. 2017 | Assesses the family’s accommodation to the child’s OCD symptoms during the previous month | Parents | 5-point Likert-scale ranging from 0 to 76, with higher scores indicating more accommodation.  0 -Never  1 = Once a week  2 = 2-3 times a week  3 = 4-6 times a week  4 = Every day |
| MASC | Multidimensional Anxiety Scale for Children | Farrell et al. 2016 | Assesses the presence of symptoms related to anxiety disorders in children and youth ages 8 to 19 years | Self-Assessment & Parents | 4-point Likert-scale:  0-Never true about me  1-Rarely true about me  2-Sometimes true about me  3-Often true about me  Maximum possible score of 117, higher scores indicating more symptoms. |
| NIMH GOCS | National Institute of Mental Health Global Obsessive-Compulsive Scale | Farrell et al. 2016 | Rates the severity of [obsessive-compulsive disorder](https://en.wikipedia.org/wiki/Obsessive%E2%80%93compulsive_disorder) symptoms on a scale from 1 (minimal symptoms) to 15 (very severe) | Clinician | A scale from 1 (minimal symptoms) to 15 (very severe) |
| PWA | Parent Working Alliance | Storch et al. 2011 | Rates parent satisfaction with services | Parents | 5-point Likert scale:  0 = very false/very dissatisfied to 4 = very true/very satisfied  Total score ranges from 0–20, with higher scores indicating stronger alliance. |
| PedsQL | Pediatric Quality of Life Scale | Farrell et al. 2016 | Measures health related quality of life in healthy children and adolescents and those with acute and chronic health conditions | Self-Assessment & Parents | Higher scores indicate better health-related quality of life. Scores range from 0 to 92 |
| SCAS-S | Spence Child Anxiety Scale short version for children/ parents | Lenhard et al. 2017  Lenhard et al. 2014 | Assesses anxiety symptoms in children | Self-Assessment & Parents | 4-point Likert-scale.  Never = 0  Sometimes = 1  Often = 2  Always = 3  Maximum possible score of 132, higher scores indicating more symptoms. |
| SDQ | Strength and Difficulties Questionnaire | Lenhard et al. 2014 | A brief behavioral screening questionnaire | Self-Assessment & Parents | Total difficulty score ranges from 0 to 40, with higher scores indicating more issues on:  1) emotional symptoms (5 items)  2) conduct problems (5 items)  3) hyperactivity/inattention (5 items)  4) peer relationship problems (5 items)  5) prosocial behaviour (5 items) |
| WAI | Working Alliance Inventory | Comer et al. 2017 | Non-standardised assessment on therapist working alliance | Self-Assessment | 8-point Likert scale. Total scores range from 36 to 252. Higher scores reflect more positive ratings of Working Alliance. |
